# A Retrospective Analysis of the Cartilage Kunitz Protease Inhibitory Proteins Identifies These as Members of the Inter-α-Trypsin Inhibitor Superfamily with Potential Roles in the Protection of the Articulatory Surface

**DOI:** 10.3390/ijms20030497

**Published:** 2019-01-24

**Authors:** Susan M. Smith, James Melrose

**Affiliations:** 1 Raymond Purves Bone and Joint Research Laboratory, Kolling Institute, Northern Sydney Local Health District, St. Leonards, NSW 2065, Australia; Susan.smith@sydney.edu.au; 2 Graduate School of Biomedical Engineering, University of New South Wales, Sydney, NSW 2052, Australia; 3 Sydney Medical School, Northern, The University of Sydney, Royal North Shore Hospital, St. Leonards, NSW 2065, Australia; 4 Faculty of Medicine and Health, University of Sydney, Royal North Shore Hospital, St. Leonards, NSW 2065, Australia

**Keywords:** serine proteinase inhibitor, Kunitz, bikunin, inter-α-trypsin inhibitor, pre-α-trypsin inhibitor

## Abstract

Aim: The aim of this study was to assess if the ovine articular cartilage serine proteinase inhibitors (SPIs) were related to the Kunitz inter-α-trypsin inhibitor (ITI) family. Methods: Ovine articular cartilage was finely diced and extracted in 6 M urea and SPIs isolated by sequential anion exchange, HA affinity and Sephadex G100 gel permeation chromatography. Selected samples were also subjected to chymotrypsin and concanavalin-A affinity chromatography. Eluant fractions from these isolation steps were monitored for protein and trypsin inhibitory activity. Inhibitory fractions were assessed by affinity blotting using biotinylated trypsin to detect SPIs and by Western blotting using antibodies to α1-microglobulin, bikunin, TSG-6 and 2-B-6 (+) CS epitope generated by chondroitinase-ABC digestion. Results: 2-B-6 (+) positive 250, 220,120, 58 and 36 kDa SPIs were detected. The 58 kDa SPI contained α1-microglobulin, bikunin and chondroitin-4-sulfate stub epitope consistent with an identity of α1-microglobulin-bikunin (AMBP) precursor and was also isolated by concanavalin-A lectin affinity chromatography indicating it had *N*-glycosylation. Kunitz protease inhibitor (KPI) species of 36, 26, 12 and 6 kDa were autolytically generated by prolonged storage of the 120 and 58 kDa SPIs; chymotrypsin affinity chromatography generated the 6 kDa SPI. KPI domain 1 and 2 SPIs were separated by concanavalin lectin affinity chromatography, domain 1 displayed affinity for this lectin indicating it had *N*-glycosylation. KPI 1 and 2 displayed potent inhibitory activity against trypsin, chymotrypsin, kallikrein, leucocyte elastase and cathepsin G. Localisation of versican, lubricin and hyaluronan (HA) in the surface regions of articular cartilage represented probable binding sites for the ITI serine proteinase inhibitors (SPIs) which may preserve articulatory properties and joint function. Discussion/Conclusions: The Kunitz SPI proteins synthesised by articular chondrocytes are members of the ITI superfamily. By analogy with other tissues in which these proteins occur we deduce that the cartilage Kunitz SPIs may be multifunctional proteins. Binding of the cartilage Kunitz SPIs to HA may protect this polymer from depolymerisation by free radical damage and may also protect other components in the cartilage surface from proteolytic degradation preserving joint function.

## 1. Introduction

Inter-α-trypsin inhibitor (ITI) was first discovered as a serum proteinase inhibitor by Steinbuch and Loeb in 1961 [1]. A series of studies subsequently demonstrated ITI in the sera of a number of animals including, bovine, cervine, equine, porcine, donkey, lapine, canine, rat and ovine sources and its susceptibility to limited proteolysis by a trypsin-like serine proteinase yielding a number of characteristic trypsin inhibitory species [2,3,4,5]. Further studies led to the identification of two Kunitz protease inhibitor domains in the ITI light chain which was subsequently named bikunin [2,5]. An additional related large ITI serum inhibitor of 120 kDa was also identified, which could be cleaved by limited digestion with kallikrein into 100 and 35 kDa fragments [6], this was named pre-α-trypsin inhibitor (pre-α-TI) [7]. Bikunin was subsequently shown to be a proteoglycan with the identification of a chondroitin sulfate (CS) side chain [8,9,10,11,12]. Bikunin is unique among the proteoglycans in that its CS chain acts as an internal linkage module for the attachment of a number of heavy chain proteins in ITI [5,13]. The mature ITI/pre-α-TI molecule contains three heavy and one light chain in a molecule of 120–250 kDa in size synthesised in the liver and secreted into serum [5]. Further studies have demonstrated up to six heavy chains attached to ITI in a number of tissues [14,15] and truncated heavy chains in fibrillated regions of osteoarthritic (OA) human articular cartilage (AC) [16].

ITI has interactive properties with hyaluronan (HA) which are promoted by the small glycoprotein TSG-6 particularly under inflammatory conditions. This may protect the HA through transfer of ITI heavy chains which cross-link the HA stabilising and countering its de-polymerisation by free radicals released by inflammatory cells during OA and rheumatoid arthritis (RA) [17]. The ITI Kunitz serine protease inhibitor (SPI) proteins detected in AC are biosynthetic products of the chondrocytes [18]. Previous studies with isolated chondrocytes and intervertebral disc (IVD) cells have demonstrated the synthesis of 6, 12, 26, 36, and 58 kDa SPI species in alginate bead culture [18,19]. How intact ITI is degraded into its Kunitz SPI domains (KPIs) in AC has not been determined but by analogy to in vitro studies on serum ITI this is likely to involve a serine proteinase, chondrocytes synthesise a chymotrypsin-like serine protease which is a likely candidate [20]. Chymotrypsin affinity chromatography of cartilage SPI-120 and SPI-58 converts these to 36, 26, 12 and 6 kDa KPI species confirming earlier ovine studies [18,21] similar KPIs have been generated from sheep serum ITI [22]. The ITI SPIs are normally identified using antibodies; however, this does not give any information on their biological status. In the course of our studies on the SPIs of AC and IVD we developed a sensitive Affinity blotting procedure using biotinylated trypsin (bT), which can be used alongside conventional antibody blots. This is a useful sensitive technique capable of detecting low ng-pg levels of SPI and also demonstrates the SPI is biologically active [21,23,24]. In the present study we have used both of these techniques which complement one another to demonstrate that many of the SPIs previously identified in AC and IVD are in fact members of the ITI superfamily [18,19,21,25,26,27,28,29,30,31].

Despite displaying potent protease inhibitory properties against a number of serine proteases including leucocyte elastase and cathepsin G, pancreatic trypsin and chymotrypsin, plasmin, kallikrein and some of the coagulation cascade proteinases the requirement for additional serine protease inhibitory activity in serum is questionable given that serum already contains α1-proteinase inhibitor, antithrombin-III, C1 esterase inhibitor, α2-antiplasmin, α1-antichymotrypsin, and α2-macroglobulin which display a broad inhibitory spectra and high capacity for protease inhibition. Thus, in some respects ITI was a redundant SPI activity in serum. On-going studies on ITI have however now shown it has a diverse range of properties far and beyond that of protease inhibition [12,32,33,34,35,36,37]. ITI transports its heavy chains to HA, a process of trans-esterification catalysed by the enzyme TSG-6 [17,38]. This produces condensed HA deposits in certain tissues of functional significance, HA deposition around oocytes is essential for their protection from free radical damage and in their expansion to promote fertilisation [39,40], condensed HA is also found in the growth plate and appears important in the cartilage to bone transition in endochondral ossification [41]. The KPI domains of ITI also have intrinsic biological properties and convey anti-bacterial [42,43], anti-viral [44,45,46,47], anti-tumour [44,45], anti-inflammatory [33,37,48,49], innate immunomodulatory properties in host-defense [50] and cell regulatory properties which promote wound repair [51]. KPI domain proteins are bi-functional in a number of marine, parasite, snake, scorpion and tick venom KPI proteins providing voltage-gated ion blocking properties as well as protease inhibition [52,53,54,55,56,57,58]. This provides clues as to the full functional capability of ITI in mammalian tissues. The present study isolated and characterised cartilage KPIs generated in-situ from a α1-microglobulin-bikunin precursor protein related to ITI and pre-α-TI. These should not be confused with two additional 6 and 12 kDa cationic SPIs found in human AC (secretory leucocyte proteinase inhibitor, SLPI; and elafin) which are also Kunitz domain SPIs but derived from different precursor proteins to the ITI SPIs [43,51,59,60]. Ovine AC also contains two homologues of SLPI and elafin (ovine SLPI and Trappin ovine molecule, TOM), which share moderate levels of sequence homology (~60%) [61,62,63].

## 2. Results

**Figure 1:**
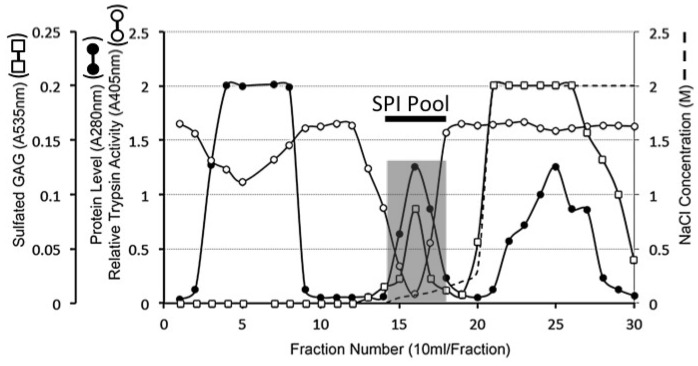
Diethylaminoethyl (DEAE) Anion exchange chromatography of a 6 M urea extract of ovine articular cartilage showing protein (A280 nm), sulfated glycosaminoglycan (A535 nm), and relative trypsin inhibitory activity of the fractions (A405 nm). The column was eluted with a gradient of NaCl (dotted line). Fractions displaying serine proteinase inhibitor (SPI) activity were pooled as shown for further purification.

Anion exchange chromatography of the 6 M urea extract of pooled ovine stifle joint cartilage resulted in ~60% of the extracted protein unbound and bound SPI which was eluted at between 0.1 to 0.3 M NaCl in fractions 13–19 (Figure 1). These SPI containing fractions were pooled for further analysis. A minor proportion of the total sulfated glycosaminoglycan (GAG) of the extract was also eluted in the pooled SPI fractions, however the majority of the sulfated GAG eluted at 2 M NaCl in the step change of the elution profile. This represented aggrecan, the major AC CS-proteoglycan (Figure 1). This initial step was useful to separate the cartilage SPI from aggrecan a major cartilage protein which otherwise interferes with further analysis of the SPIs.

**Figure 2:**
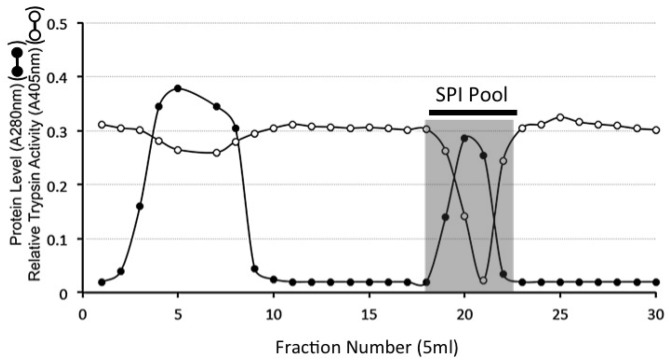
Hyaluronan (HA) affinity chromatography of the serine proteinase inhibitor (SPI) pool from DEAE Anion exchange chromatography of the ovine AC SPIs. The column was eluted with 2 bed volumes of starting buffer 50 mM Tri-HCl pH 7.2 and the bound SPI eluted by a step change elution with 2 M NaCl in starting buffer. Fractions containing trypsin inhibitory activity were pooled (SPI pool) for further analysis.

The DEAE SPI pool was next subjected to HA affinity chromatography (Figure 2). A small proportion of SPI and major proportion of protein did not bind under the chromatographic conditions used however the majority of the extracted AC SPI displayed an affinity for HA and was subsequently eluted with 2 M NaCl (Figure 2).

**Figure 3:**
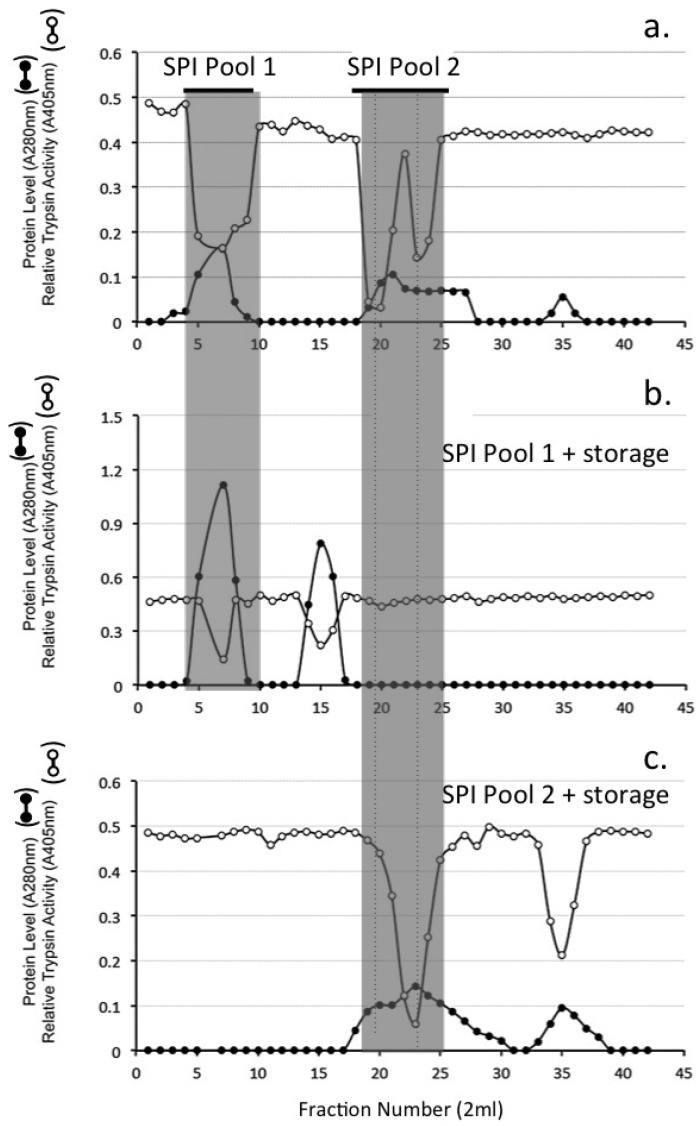
Sephadex G100 gel permeation chromatography of the ovine articular cartilage (AC) SPI sample from HA affinity chromatography (**a**) Fractions were monitored for protein (A280 nm) and relative trypsin inhibitory activity (A405 nm). Fractions containing trypsin inhibitory activity were pooled. Two SPI pools were collected as shown and separately re-chromatographed on the same column after 2 weeks storage at 4 °C (**b**,**c**).

Sephadex G100 gel permeation chromatography of the HA affinity bound SPI resulted in the elution of ~30% of the SPI in the void volume of the column (Fraction 4–8) while a larger proportion of the SPI eluted well included into the column over fractions 18–26 (Figure 3a). These SPI containing fractions were separately pooled (SPI pool 1 and SPI pool 2) and stored at 4 °C for one month prior to re-chromatography on the same column (Figure 3b,c). The void volume SPI pool sample eluted as two discernable SPI peaks at the void volume and also in fractions 12–16 (Figure 3b). The SPI pool 2 sample from the initial separation on Sephadex G100 (Figure 3a) eluted over a similar size range after storage however the SPI activity profile of this peak was skewed towards a smaller molecular weight range and an additional SPI peak was evident in fractions 32–38 (Figure 3c).

**Figure 4:**
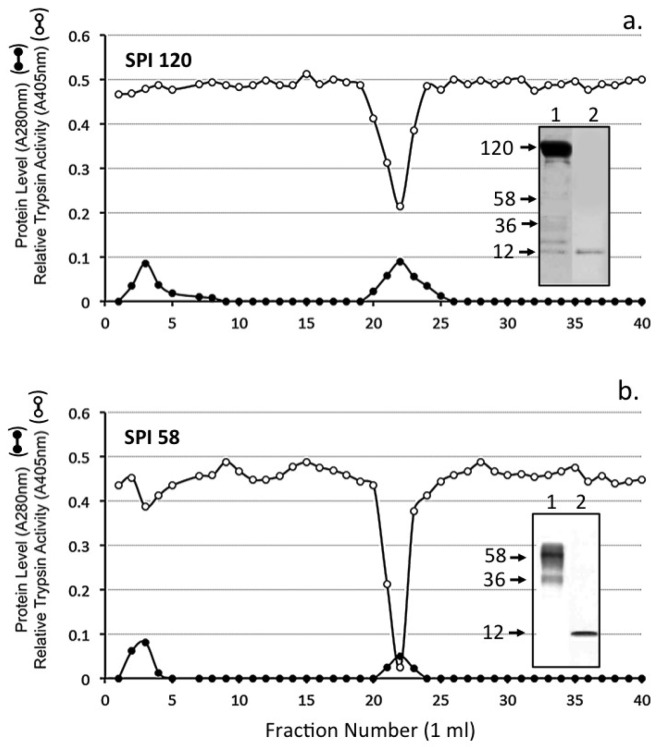
Chymotrypsin affinity chromatography of the SPI 120 (**a**) and SPI 58 (**b**) SPI pools 1 and 2 from Figure 3a. The insets depict biotinylated trypsin (bT) affinity blots, which identify active SPIs in the sample prior to (1) and after (2) chymotrypsin affinity chromatography.

Samples of the SPI pools 1 and 2 from Sephadex G100 gel chromatography of the ovine AC SPI (Figure 3a) were subjected to chymotrypsin affinity chromatography. All detectable SPI activity was bound to this affinity matrix and eluted over fractions 20–25 (Figure 4). Affinity blotting using bT of the Sephadex G100 SPI pool 1 and 2 samples prior to and after chymotrypsin affinity identified major 120 kDa SPI (SPI120) and 36–58 kDa SPIs (SPI58) which were converted to a 12 kDa SPI species by the affinity procedure (Figure 4 insets).

**Figure 5:**
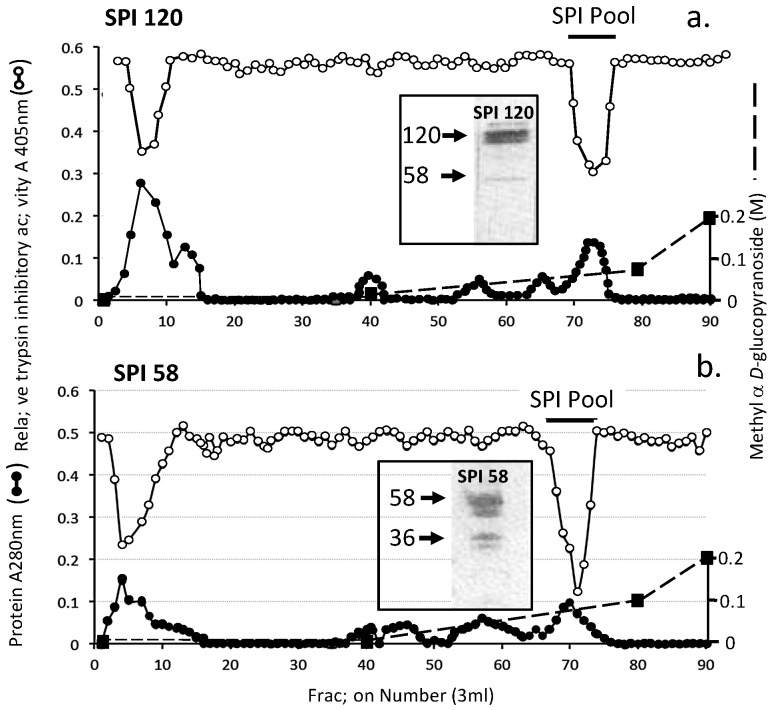
Concanavalin A affinity chromatography of SPI 120 (**a**) and SPI 58 (**b**) SPI pool 2 Figure 3a. The SPI58 pool was applied and the column eluted with 10 bed volumes of starting buffer (50 mM Tris, 200 mM NaCl buffer pH 7.2 starting buffer containing 1 mM CaCl_2_, 1 mM MgCl_2_, and 1 mM MnCl_2_). Bound material was eluted with a linear 0–0.2 M gradient of methyl α-glucopyranoside as shown. The inset shows active 58 and 36 kDa SPIs on bT affinity blots of the SPI58 sample prior to and after Concanavalin A affinity chromatography.

Concanavalin A lectin affinity chromatography of the SPI58 sample from Sephadex G100 chromatography (SPI Pool 2, Figure 3a) resulted in the isolation of a bound SPI pool containing a closely spaced SPI doublet (45–58 kDa) and minor 36 kDa species (Figure 5).

**Figure 6:**
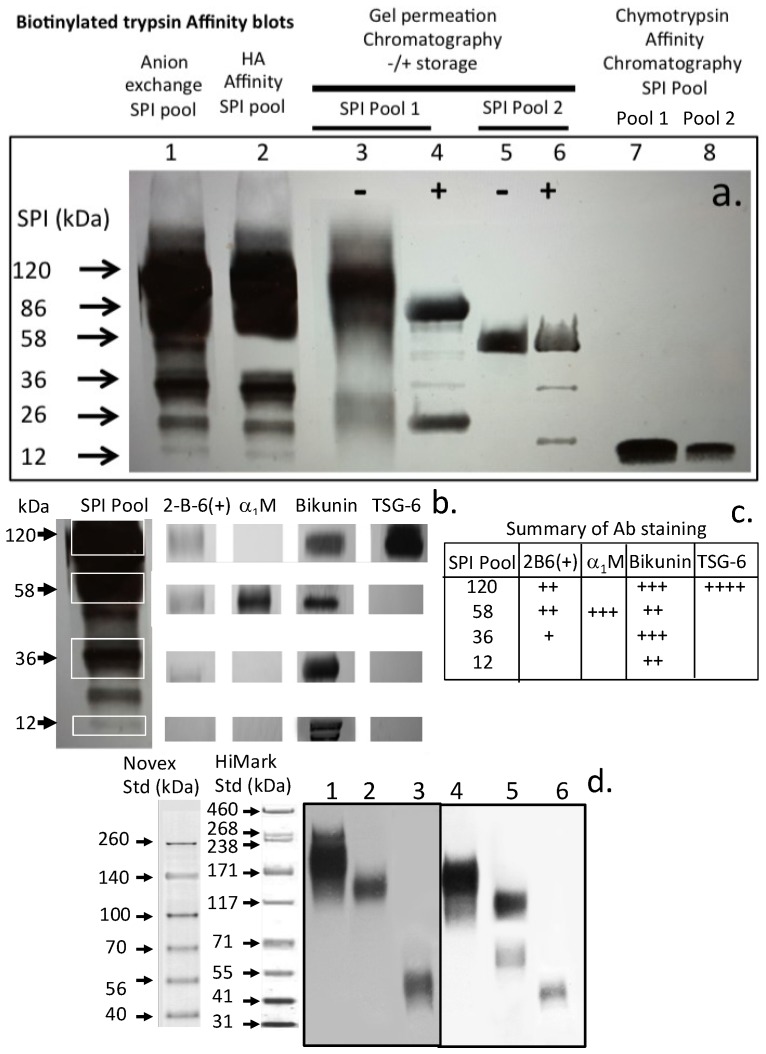
Identification of serine proteinase inhibitor (SPI) species using biotinylated trypsin and avidin alkaline phosphatase conjugate by affinity blotting in samples from the various isolation procedures used in this study (**a**) Selected bands were also identified using antibodies to CS (2-B-6 (+)), α1 M, Bikunin, and TSG-6 (**b**) and the blot intensities summarized in the Table in (**c**). ITI and pre-α-TI present in ovine plasma (lanes 1, 2, 4, 5) and UTI in urine (lanes 3, 6) were also identified by Western blotting using antibodies to ITI (lanes 1, 2) and bikunin (lane 3) and by affinity blotting using bT (lanes 4–6) (**d**). The extra band in lane 5 but not in lane 2 in segment (**d**) is due to α1-proteinase inhibitor which also displays an affinity for HA thus is also included in the pre-α-TI pool taken from Sephadex G200 chromatography in our isolation scheme.

**Figure 7:**
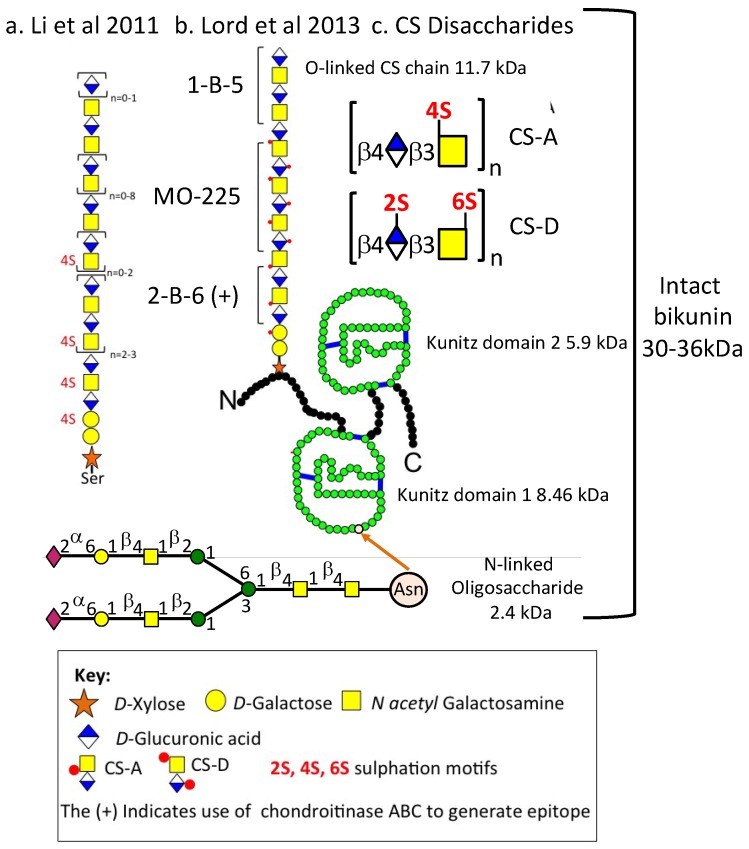
Sequence of the CS chain of bikunin as proposed by Ly et al. 2011 [11] (**a**) and Lord et al. 2013 [10] (**b**). The KPI SPI domains are shown in (**b**). and the attached *N*-glycan chain of KPI-1. The over-sulfated CS-D disaccharide is shown which forms part of the MO-225 epitope embedded in the CS-A chain of bikunin. Arrangement of sulfated, disulfated and non-sulfated regions of the CS chain of bikunin are shown. The arrow shows the Asparagine residue on Kunitz domain 1.

**Figure 8:**
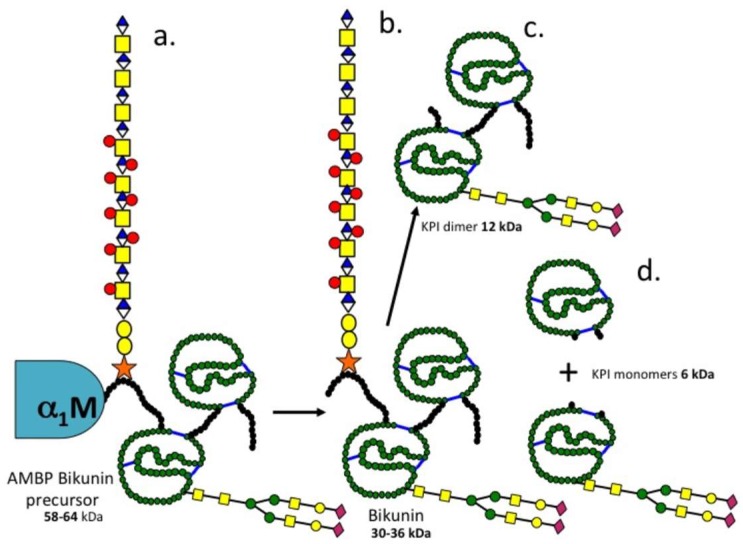
Schematic representations and proposed inter relationship between SPI species seen in this study. a. α1 microglobulin-bikunin precursor (58–64 kDa), b. bikunin 30–36 kDa, c. bikunin KPI dimer (12–16 kDa) after removal of the 11.7 kDa CS chain from bikunin, d. bikunin KPI monomers (6–9 kDa) with the N-linked 2–4 kDa glycan oligosaccharide attached to the KPI-1 domain which facilitates its isolation by ConA affinity chromatography and the 6 kDa KPI-2 domain which does contain oligosaccharide substitution.

Affinity blotting of SPI samples from various stages of the isolation procedure, (1) anion exchange, (2) HA affinity, (3–6) Sephadex G100 chromatography SPI pools 1 and 2 ± storage at 4 °C and [7,8] after chymotrypsin affinity identified a range of SPI species of molecular weight 120, 86, 58, 36, 26 and 12 kDa (Figure 6a). Western blotting using antibodies to the 2-B-6 stub epitope of the CS side chain of bikunin. α1-microglobulin, bikunin and TSG-6 identified the molecular organisation of these SPIs. Selected regions of replicate blot lanes from anion exchange isolated ovine cartilage SPIs were cut into regions corresponding to the 120, 58, 36 and 12 kDa SPI bands and examined using antibodies to the chondroitin-4 sulfate stub epitope of CS chains (2B6 (+)), α1-microglobulin (α1-M), bikunin and TSG-6. The blot segments for the 2B6 (+) determinations were digested overnight with chondroitinase ABC during the blocking step. This removes the CS chains generating a 2B6 (+) stub epitope which identifies CS substitution on that SPI species which migrates with the position of the intact CS-SPI during SDS PAGE. A summary of the antibody staining’s for each SPI band is provided in Table C. Comparison of a bikunin blot (lanes 1–3) and bT affinity blot (lanes (4–6) of serum, plasma and urine samples. The smaller SPI species in lane 5 not detected in lane 2 is due to α1-proteinase inhibitor which binds to the HA affinity column during the isolation steps. Diagrammatic representations of the structural organisation of bikunin/ITI and the deduced inter-relationships between the KPIs identified in the present study are presented in Figure 7 and Figure 8.

Assessment of the inhibitory activity of the isolated KP1 and KP2 ITI SPI domains against a range of serine proteinases using specific anilide substrates demonstrated these had potent inhibitory activity against porcine pancreatic trypsin and chymotrypsin, human leucocyte elastase and cathepsin G, porcine kallikrein and plasmin comparable to BPTI (Table 1). KPI-1 was less inhibitory against human leucocyte cathepsin G, porcine kallikrein and plasmin compared to BPTI however still displayed appreciable inhibitory activity against these proteinases (Table 2).

**Figure 9:**
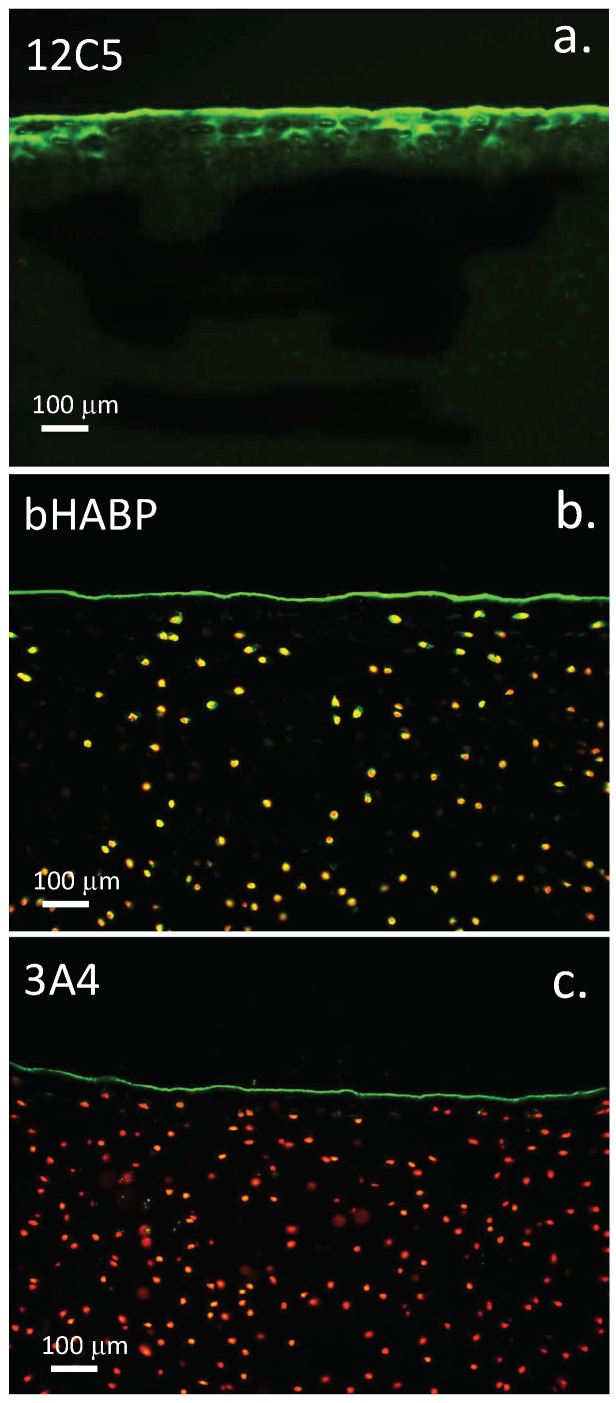
Immunolocalisation of versican in the surface regions of tibial plateau bovine articular cartilage (**a**) immobilises HA at the cell surface (**b**). This is visualised using biotinylated HABP and avidin-FITC. Lubricin is also a component of the surface lamina of articular cartilage (**c**) and has roles in joint lubrication acting synergistically with HA and other proteins such as fibronectin and pentraxin-3 which aid in joint lubrication. ITI SPIs are also attached to HA and these protect the cell surface lubricin. HA is also visualised intra- and pericellularly in the articular chondrocytes (**b**). Cell nuclei were stained with propidium iodide in b and c. HA was visualised using bHABP/avidin-FITC. The fluorescent images were visualised by confocal microscopy using a Leica TCS SP2 AOBS laser scanning confocal microscope using a 40× oil immersion objective. The slides were scanned using excitation and emission settings for Alexa 488 of (Ex max: 488; Em max: 520) Z-stacks of 8-bit ‘optical sections’ (512 × 512 pixels) were taken through the full cartilage thickness at in 0.4 μm increments. ‘Maximum Intensity’ reconstructions were prepared from the image stacks using Leica Confocal Software (Leica, Heidelberg, Germany).

Versican was prominently immunolocalised in the surface regions of bovine knee articular cartilage, as was PRG4 (Figure 9a). Biotin labelled hyaluronan binding protein (bHABP) was used to localise HA, this was visualised using an avidin-FITC secondary reagent (Figure 9b). Lubricin was immunolocalised using MAb 3A4 and an Alexa 488 conjugated secondary antibody (Figure 9c).

## 3. Discussion

### 3.1. Identity of the Ovine Cartilage SPIs

The present study has identified a 58 kDa α1-microglobulin-bikunin precursor protein (SPI 58) which was converted to a number of smaller SPIs either by prolonged storage or by chymotrypsin affinity chromatography [18,21]. A 120 kDa SPI was also detected which had the CS attachment stub epitope identified by MAb 2-B-6 (+) and was also reactive with antibodies to bikunin and TSG-6 consistent with its identity as pre-α-TI. All of the SPIs generated from SPI 58 were reactive with an antibody to bikunin. A canine IVD study has previously identified 120–250 kDa SPIs cross-reactive with an ITI antibody [31]. Pre-α-TI is susceptible to cleavage by kallikrein into 100 and 35 kDa fragments [6] and trypsin also degrades ITI into a number of characteristic fragments of similar size to those seen in the present study [64]. The ovine cartilage SPI 58 and 120 was also isolated by concanavalin A lectin affinity chromatography confirming *N*-glycosylation known to be present on bikunin. SPI 26 was also isolated using concanavalin A lectin affinity chromatography, SPI-26 was also reactive with anti-bikunin but not antibodies to 2-B-6 (+), α1-M or TSG-6 thus identifying this as a KPI dimer devoid of the CS chain of bikunin.

### 3.2. The Structural Organisation of Bikunin

Bikunin is the simplest proteoglycan known, consisting of a 146 amino acid core protein, which is synthesised as a 337 amino acid 58 kDa α1-microglobulin-bikunin precursor protein (AMBP) which is cleaved at Arg-186 to release α1-microglobulin and bikunin. Bikunin is glycosylated at Ser-10 with a single heterogeneous *O*-linked 15–25 kDa chondroitin-4-sulfate chain containing 23–55 saccharide residues; a biantennary *N*-linked glycan is also attached at Asn-45. The 16 kDa bikunin core protein is organised into two 6 kDa Kunitz protease inhibitor domains which are each stabilised by three internal disulphide bonds [65]. The schematic in Figure 7 depicts the CS side chain organisation and its attachment to bikunin as proposed by Ly et al. [11], Lord et al. [10] and Fries and Blom [32]. Ly et al. identified the consensus sequence of the CS-A chain of bikunin concluding that the identification of the precise sequence of this CS chain was a difficult proposition due to its micro-heterogeneity. Lord et al. subsequently went on to demonstrate embedded CS-D disaccharides within the CS-A chain using monoclonal antibody MO-225, these are considered important for the interactive properties of the bikunin CS chain [10]. The general depiction of the bikunin molecule by Fries and Blom [32] shows the *O*-linked CS chain in the N-terminal extension peptide of KPI domain 1 and *N*-glycosylation site at Asn-45 [10,11]. 

### 3.3. Inter-Relationships between Ovine Articular Cartilage SPI Species and Bikunin/ITI

Comparison of data generated in the present study with published data on ITI fragments generated by trypsin or kallikrein digestion allowed us to interpret inter-relationships between the ovine AC SPIs observed in the present study. A diagram of the deduced structures of these SPIs is presented in Figure 8. SPI58 was detected using antibodies to α1-microglobulin, bikunin and 2-B-6 (+) CS stub epitope thus represented the CS substituted AMBP-bikunin precursor protein. Chymotrypsin affinity chromatography generated 12 and 6 kDa SPIs from SPI58. This protein appears quite labile and prolonged storage of SPI58 at 4 °C also generated 36, 26 and 12 kDa KPI species, these were identified by a polyclonal anti-bikunin antibody, SPI36 contained a 2-B-6 (+) reactive CS epitope demonstrating the presence of a CS side chain while SPI26 did not, but contained variable portions of the N and C terminal peptide extensions attached to the KPI dimer. We also deduced that SPI12 represented the double headed KPI devoid of these peptide extensions. Furthermore, SPI6 could be generated by chymotrypsin affinity representing individual KPI domains 1 and 2, these were separable from one another using concanavalin-A lectin chromatography on the basis of the absence or presence of *N*-glycosylation. 

### 3.4. ITI/Bikunin Is A Multifunctional Protein

Despite its discovery almost six decades ago the primary role of ITI has been elusive. Subsequent studies have established functional roles for the ITI HC and KPI domains in their own right. While the KPI domains of the bikunin light chain of ITI confer protease-inhibitory properties they also provide anti-tumour and anti-viral properties. Furthermore, the HCs attached to the CS chain of bikunin mediate ITI’s protein-protein interactions with many ECM components. TSG6 is one such interactive protein, which promotes the transfer of the ITI HCs to HA, cross-linking and stabilising HA through a unique trans-esterification reaction [66]. In some cases, the accumulation of HA-HCs in tissues can be beneficial such as in the ripening and fertilisation of the oocyte or the maturation of the growth plate cartilages during endochondral ossification [39,40,41,67]. In other cases, HC-HA accumulation in a condensed form in tissues hampers tissue function. Besides AC, ITI is also expressed in brain, placenta, liver, heart, lung, kidney and IVD and may be of importance in organ development [19,38]. Several observations suggest that like other members of the KPI superfamily, bikunin has anti-tumour and anti-viral activities, expression of bikunin by human glioblastoma cells suppresses tumour invasion [68] and addition of bikunin to human chondrosarcoma cell cultures blocks cell spreading [69]. Prior to this, low molecular weight factors had been detected in pregnant urine and pharmaceutical grade preparations of human chorionic gonadotrophin (hCG) which actively inhibited Kaposi Sarcoma (KS) lesion development in HIV infections. These factors were initially termed antiviral lysozyme-C or antiviral RNases [70,71,72,73,74]. The appreciation that bikunin possessed anti-viral activity became apparent when a 15.8 kDa fragment of bikunin was identified as a contaminant in hCG preparations which inhibited the spread of KS lesions in HIV infections [75].

### 3.5. Detrimental Aspects of HC-HA Complex Transfer in Tissues

During inflammation and developmental processes, HCs from ITI are covalently transferred to HA via the enzyme TSG-6 to form an HC-HA complex. This is a significantly more adhesive substrate for leukocytes than non-cross-linked HA, and can enhance inflammation in pathological conditions. The accumulation of pro-inflammatory HC-HA in lung tissue in cystic fibrosis exacerbates chronic inflammation in airway disease and increases mucus viscosity making it difficult to eliminate from the bronchioles reducing O_2_ transfer and impairing lung function [76]. Hyperglycemia induces accumulation of HA around vascular smooth muscle cells, increases aortic stiffness and strength, and primes the vascular wall for the deposition of cholesterol, accumulation of leucocytes and accelerated development of atherosclerosis in ApoE^−/−^ mice [77]. Accumulation of HA and HC-HA in lung tissue correlates with impaired lung function in asthma and the inflammation of lung tissues [78]. With the development of type I diabetes, HA is dramatically increased within and outside the islet pancreatic endocrine cells, contributing to the pathogenesis of diabetes [79]. ITI HC proteins accumulate in tumours and are implicated in their pathogenesis [80,81]. Renal epithelial cells produce bikunin when stimulated by nephrotoxic agents such as oxalate [82], HA also inhibits calcium oxalate crystallisation in vitro [83]. Urinary trypsin inhibitor (UTI) has a regulatory effect on local vascular tone by regulation of Ca2+ influx suppressing smooth muscle contraction [84] and also prevents lipopolysaccharide-induced increases in cytosolic free Ca2+ in human neutrophils and human umbilical vein endothelial cells [85]. 

The KPI proteins are a diverse group of proteins occurring in all phyla in nature. Many invertebrate KPIs display blocking properties for voltage gated ion-channels. The parasitic worm *Echinococcus granulosus* synthesises a KPI peptide that blocks cation channels [86]. ShPI-1 and APEKTx1, BPTI-like KPIs from the sea anemones *Stichodactyla helianthus* [53] and *Anthopleura elegantissima* [55] and the anemone toxins kalicludines and kaliseptine are homologous to snake venom dendrotoxins and also display ion-blocking properties [56]. Calcicludine, a venom peptide of *Dendroaspis angusticeps*, homologous to snake dendrotoxins and the KPI APP/protease nexin-2 domain of the brain proteoglycan appican [54,87] blocks high-threshold Ca2+ channels in cerebellar granule neurons [57] and homologous to LmKTT-1a a bifunctional KPI and potassium channel blocking scorpion peptide toxin [88]. The snake toxin BF9 also displays KPI activity and potassium channel inhibitory activity [58]. These ion-blocking properties are related to the neuroregulatory properties displayed by bikunin and appican in the CNS.

### 3.6. Tissue Isoforms of ITI

Serum ITI has historically been depicted as a molecule containing two heavy chains (HC1 and HC2) while pre-α-TI has another HC (HC3) however up to six HCs can be attached to the CS chain of bikunin/ITI with tissue development and pathology [89]. A systematic review of gene and transcript expression profiles using microarray and sequencing based functional genomics and antibody-based profiling [90,91,92,93,94] is working towards a full description of a tissue-based map of the human proteome [95]. Thus, a comparison of the expression profiles of the *ITIH1–ITIH5* genes in tissues demonstrates that *ITIH1–ITIH4* are predominantly expressed in liver while *ITIH5* is expressed in breast, skin, adipose tissue and placenta [15,89]. *ITIH5* is over-expressed in inflammatory skin diseases such as psoriasis, atopic dermatitis and allergic contact dermatitis [15] and specifically in the suprabasal layers of the epidermis. *ITIH5* is also expressed by normal skin fibroblasts but not by epidermal keratinocytes [89] and is a novel putative tumour suppressor gene in colon cancer [96]. *Renal ITIH3* expression may regulate oxalate kidney stone formation [83]. HA also inhibits calcium oxalate crystallisation in vitro [83]. Novel truncated 50 kDa forms of HC1 and HC2 have been detected in OA AC [16], full length 90 kDa HCs attached to HA were also observed in the synovial fluids of OA patients. Bikunin and ITI are abundant in regions of surface fibrillation in OA AC.

### 3.7. Beneficial Aspects of HC-HA Transfer in Connective Tissues

Mesenchymal stem cells (MSCs) are pluripotent, differentiating into osteoblasts, chondrocytes, and adipocytes in vitro and in vivo. Umbilical MSCs (UMSCs) exposed to inflammatory cells synthesise an extracellular glycocalyx rich in HA bound to ITI HCs and the enzyme TSG6 which catalyses the transfer of HCs to HA, versican, and pentraxin-3 [97]. This glycocalyx regulates inflammatory cells and allows UMSCs to survive host immune rejection. Furthermore, the focal up-regulation of HA and ITI in areas of muscle damage and the temporal acute expression of TSG6 by MSCs is conducive to the creation of a microenvironment favoring the engraftment of MSCs in areas of damaged muscle promoting tissue repair [98]. 

### 3.8. Protective Roles for ITI KPIs in Connective Tissues

MMPs have important roles in tissue remodeling in physiological and pathological conditions in tissue development, morphogenesis, angiogenesis, tissue repair, arthritis and in tumour development. AC SPIs have roles in the prevention of excessive degradation of ECM components following traumatic overload in post-traumatic OA or in the inflammatory conditions of RA. Kunitz domain 2 of ITI (trypstatin) is taken up by mast cells and is found complexed with serine proteases in intracellular granules [99].

### 3.9. KPIs in Meniscus, AC and IVD

The human, canine and ovine IVD contain both low molecular weight (12 kDa) SPIs and 120 and 250 kDa ITI-like SPIs [28,31] active against human leucocyte elastase (HLE), cathepsin-G, chymotrypsin and trypsin, urokinase, plasmin, kallikrein [100], similar SPIs have been identified in costal and AC and fibrocartilaginous meniscus [25]. N-terminal sequencing demonstrated identical amino acid sequences for the cartilage and IVD SPI with SLPI from parotid and seminal plasma secretions [25]. IVD cells and articular chondrocytes also synthesised mRNA to SLPI [59]. Affinity blotting with bT [24], a solid phase enzyme linked immunofiltration assay to SLPI [101] and competitive inhibition assay developed to quantitate SPIs in normal and degenerate human IVDs [30], demonstrated depleted levels of active IVD SPI with advancing IVD pathology. Studies on canine IVD SPIs in chondrodystrophic (ChD) and non-chondrodystrophic (non-ChD) breeds [31] have differing rates of disc degeneration and an age dependent decline in active IVD SPI levels in the ChD (but not the non-ChD) breeds. BPTI has similar electrophoretic properties to the 6 kDa ovine AC SPI [21]. A chicken anti-BPTI IgY demonstrated homologies between the ovine SPIs and BPTI [28] and immunolocalised these in mast cells and chondrocytes in ovine and bovine lung and AC [29]. BPTI cross-reactive SPIs were synthesised by ovine AF and NP cells in alginate bead culture with a 6 kDa SPI secreted into the culture media and a 34–36 kDa SPI was retained within alginate beads [19]. Ovine chondrocytes also synthesised ^14^C-lysine-6 and 58 kDa SPIs in alginate bead culture [18]. A biotinylated potato chymotrypsin inhibitor affinity probe demonstrated that chondrocytes synthesised an active ^14^C-chymotrypsin-like serine proteinase in alginate bead culture [20], which may generate the 6 kDa ovine Kunitz SPI from the 58-kDa SPI precursors.

### 3.10. Bikunin As A Cell Regulatory Proteoglycan

Oversulfated CS/DS promotes neuritogenesis and regulates CNS development. The disulfated disaccharide CS-D-unit, promotes neurite outgrowth through the DSD-1 epitope embedded in the CS chains of DSD-1-PG/phosphacan [100,101,102,103,104,105,106]. Over sulfated DS also exhibits neurite outgrowth activity [107], the short isoform variant of phosphacan/receptor protein tyrosine phosphatase-β, interacts with neuronal receptors and promotes neurite outgrowth [108]. Bikunin is also expressed in brain tissue [107,109] and accumulates in brain tumours [81]. Like phosphacan, bikunin contains embedded CS-D motifs within the repeat disaccharide region of its CS chain. Such motifs in phosphacan promote neurite outgrowth suggesting that bikunin may also have roles to play in neural development. Appican is another CS brain proteoglycan containing embedded CS-E residues within its CS side chains [87,110] and is produced by astrocytes [111]. These CS-E motifs [112] interact with neuroregulatory factors [113] inducing morphological change in C6 glioma cells and directed adhesions of neural cells to the ECM [114] and also promote the chondrocytic differentiation of ATDC5 cells [115]. HC chain transfer from the bikunin CS chain provides matrix stabilisation, oocyte expansion and is essential in fertilisation but CS-HCs may affect HA turnover adversely and have deleterious effects on physiological processes in cystic fibrosis, diabetes, asthma, hyperglycemia, tumour development and atherosclerosis. Microarray analysis has identified a number of bikunin target genes in ovarian cancer cells and these have been categorised as transcriptional regulators, oncogenes/tumour suppressor genes, signaling molecules, growth/cell cycle, invasion/metastasis, cytokines, apoptosis, ion channels, extracellular matrix proteins, as well as some proteases [116]. This further emphasises bikunin as a multifunctional cell regulatory proteoglycan.

### 3.11. Localisation of HA and ITI SPIs at the Articular Surface Is of Physiological Significance

Versican is found localised at the surface of AC where it localises HA. As shown in this study, the ITI SPIs share an affinity for HA thus we may deduce that these would also localise in the surface regions of AC where they would protect the AC from proteolytic damage from serine proteinases. The ITI SPIs have broad inhibitory activity against a range of serine proteinases (Table 1 and Table 2) some of which have MMP activating activity, another class of cartilage degradative proteinase. The ITI SPIs also display potent ant-bacterial, ant-fungal and anti-viral activities further expanding putative biological protective roles in cartilaginous tissues.

Lubricin (PRG4) was also shown to be a component of the cartilage surface in the present study (Figure 9c); lubricin acts synergistically with HA (Figure 9b) to provide elastoviscous properties, which are important for the articulatory properties of articular cartilage [117]. HA in isolation is a relatively poor boundary lubricant. Localisation of the ITI SPIs with HA in the articular surface may protect lubricin in the surface lamina and make important contributions to the preservation of joint function. A number of catabolic enzymes have been observed which digest lubricin [118,119,120] and can be inhibited by ITI (Table 1). ITI accumulates in HA depositions in skin in a condition known as lichen sclerosis [121]. HA is depleted in AC in human OA and in a mouse model of human mucopolysaccharidosis IX which displays similar cartilage changes as found in OA [122]. In normal AC, HA is found localised to the articulatory surface regions and in the epiphyseal growth plate where it has roles to play in endochondral ossification. HA is also localised pericellularly and intracellularly in hypertrophic cells of the vertebral growth plate [123]. TSG-6 catalyses the transfer of HCs to HA, and may also transfer these to versican at the articular cartilage surface (Figure 9). ITI SPIs are also components of glycocalyx formations in the corneal surface under inflammatory conditions [124]. ITI isoforms attached to the articular surface may be attached to versican at the articular surface [16]. TSG-6 also catalyses the transfer of HC chains from ITI to stabilise amniotic membranes [125]. Pentraxin-3-HC-HA complexes have anti-angiogenic properties and roles in innate immunity complementing lubricin-TLR-2 and TLR-4 interactions in the cartilage surface to regulate inflammatory processes and maintain cartilage homeostasis [126,127]. Pentraxin-3 is a soluble pattern recognition receptor with roles to play in innate immunity [128,129]. High molecular weight HA also has anti-inflammatory properties [130]; its localisation at the cartilage surface contributes to joint lubrication. Lubricin in the surface regions of cartilage also binds to a number of cartilage proteins [131] contributing to joint lubrication, and the retention of lubricin at the cartilage surface [132] and may promote cartilage regeneration [133,134,135].

## 4. Materials and Methods

The sources and quality of reagents used in this study were as noted earlier [18,20,21]. Chondroitinase-ABC from Proteus vulgaris (Catalogue # C3667), bovine pancreatic trypsin type XI, Porcine pancreatic trypsin and chymotrypsin, porcine kallikrein and porcine plasmin, human plasma α1-proteinase inhibitor were purchased from Sigma-Aldrich (Sydney, Australia). 1,9-dimethyl methylene blue was obtained from Sigma-Aldrich, Sydney, Australia. Nitroanilide chromogenic protease substrates were purchased from Bachem Feinchernikalien AG (Bubendorf, Switzerland). BPTI as Trasylol^®^ (Aprotinin) was obtained from Bayer Healthcare through our in-house pharmacy (St Leonards, Australia). Human leucocyte elastase and cathepsin G were isolated from out of date neutrophil buffy coat preparations obtained from our blood bank (Sydney, Australia) as specified earlier [136,137]. Antibodies to α1-proteinase inhibitor (ab9373) and inter-α-trypsin inhibitor (ab204513), rabbit polyclonal anti-bikunin antibody (ab43073) were purchased from Abcam, Cambridge, MA, USA. Affinity purified mouse monoclonal antibody to TSG-6 (Catalogue # AF2326) and mouse anti-human α1-microglobulin (Catalogue # MAB7724) were obtained from R & D systems, In Vitro Technologies Pty Ltd., Melbourne, Vic, Australia. 2-B-6 Hybridoma conditioned medium and anti-lubricin (MAb 3A4) were gifts from Prof B. Caterson, University of Cardiff, Cardiff, UK. MAb 12C5 to versican G1 domain, was purchased from the Developmental Studies Hybridoma Bank, University of Iowa, Iowa city, IA, USA. Hyaluronan binding protein (HABP) was isolated from bovine nasal cartilage, biotinylated and used as indicated earlier [138,139].

### 4.1. Tissues

Ovine stifle joints were harvested from 2-year-old control merino wethers from another project, which was being undertaken. The University of Sydney Animal Care and Ethics Committee approved this study under ethics approval A45/6-2011/3/5544 (approved on 1 January 2014) and the ethical permission in this project covered the use of ovine control tissues in other projects such as the present study. Bovine knees were obtained from a local abattoir.

### 4.2. Visualisation of Cartilage Surface Components

Versican, HA and lubricin were visualised in the surface regions of bovine articular cartilage using antibody 12C5 to the G1 domain of versican, biotinylated HABP was used as described earlier to visualise surface HA deposition [125] and lubricin identified using antibody 3A4. 

### 4.3. Preparation of Biotinylated Trypsin

Biotinylated trypsin (bT) was prepared as noted earlier [24] and subjected to a final clean up step on a SBTI Affinity column to ensure a fully active enzyme preparation of high specific activity, this was aliquoted and stored frozen and its activity determined by active site titration (−20 °C).

### 4.4. Preparation of Immobilised Hyaluronan

A column of immobilised HA was prepared as outlined earlier [138,139].

### 4.5. Determination of Trypsin Inhibitory Activity

Aliquots (50 µL) of chromatographic fractions were added to individual wells of a flat bottom 96 well microtitre plate followed by assay buffer (50 mM Tris-HCl pH 8.2 containing 10 mM CaCl_2_). Trypsin (20–50 ng) in assay buffer (50 µL) was then added with mixing. The assay was initiated by addition of substrate (50 µL of CBZ-Arg-4-nitroanilide in 100% DMSO) and the plates incubated at 37 °C for 3–5 h. The plates were then read at A405 nm using a plate reader. Trypsin inhibitory activity was determined from the drop in A405 nm compared to control wells containing trypsin but no chromatographic fraction.

### 4.6. Measurement of the Relative Protease Inhibitory Activity of Bikunin KPI-1, KPI-2 and BPTI

Trypsin (EC 3.4.21.4, Sigma type XIII from bovine pancreas) treated with *N*-tosyl-phenylalanine chloromethyl ketone to inactivate any contaminating chymotrypsin activity was assayed with the substrate *N*-α-carbobenzoxy-l-arginine-p-nitroanilide (ZAPNA) [140]. The level of active trypsin was measured using the active site titrant *N*-α-carbobenzoxy-L-arginine p-nitrophenyl ester [141] and used in turn to standardise the KPI-1, KPI-2 and BPTI samples. Chymotrypsin (EC 3.4.21.1, type II from bovine pancreas) was assayed using the substrate Ala-Ala-Val-Ala 4-NA (AAVANA), human neutrophil cathepsin G (EC 3.4.21.20) activity was assayed with *N*-Succinyl-Ala-Ala-Pro-Phe 4-NA (SAAPPNA). Plasma kallikrein (EC 3.4.21.34) was assayed using d-Val-Leu-Arg 4NA and plasmin (EC 3.4.21.7) was assayed using d-Val-Leu-Lys 4NA. Human leucocyte elastase (HLE) (EC 3.4.21.37) was assayed with *N*-α-succinyl-alanyl-alanyl-valine p-nitroanilide (SAAVNA) [142]. These assays were performed in 50 mM Tris HCI, 100 mM NaCI, 20 mM CaCI_2_, 0.1 mg/mL of bovine serum albumin (BSA), and 0.02% (*w*/*v*) NaN_3_, pH 8.2. Cathepsin G (EC 3.4.21.20) was isolated as previously described [136,137], and along with commercial chymotrypsin (EC 3.4.21.1, type II from bovine pancreas) was assayed in 100 mM Tris HCI, 100 mM NaCI, 10 mM CaCI_2_, 0.1 mg/mL of BSA, and 0.02% NaN_3_ pH 7.5, using *N*-α-succinyl-alanyl-alanyl-prolylphenylalanine-p-nitroanilide (SAPA) as substrate. Assays were performed with substrate concentrations of 0.2 mM in 4% (*v*/*v*) DMSO at 37 °C in 96-well microplates in a final reaction volume of 250 µL. Absorbance values at 405 nm were recorded, using the absorbance at 690 nm as a zero reference value.

### 4.7. Extraction of Serine Proteinase Inhibitory Proteins from Articular Cartilage

Finely diced articular cartilage from eight stifle joints of 2-year-old control merino wethers (18 g wet weight) was extracted with 200 mL of extraction buffer (6 M Urea 50 mM Tris-HCl pH 7.2) with constant end-over-end mixing at 4 °C for 72 h. The tissue residue was then separated by filtration through muslin and the extract clarified by centrifugation (16,000× *g* for 20 min).

### 4.8. DEAE Sepharose 4B Anion Exchange Chromatography

Aliquots of the tissue extract (50 mL) were applied batch-wise to a 60 mL bed volume column of DEAE Sepharose at a flow rate of 10 mL/h and the column eluted with starting buffer 50 mM Tris-HCl pH 7.2 (150 mL). Bound material was eluted with a gradient of 2 M NaCl in starting buffer as follows, (i) a linear gradient from 0–0.4 M NaCl (50 mL), (ii) stepwise change to 2 M NaCl (100 mL). Eluant fractions of 10 mL were collected; aliquots of fractions (50 µL) were monitored for relative trypsin inhibitory activity and sulfated GAG content by reaction with 1,9-dimethyl methylene blue. Protein was monitored by the A280 nm of the fractions. SPI containing fractions 14–18 were subsequently pooled and further purified by HA affinity chromatography.

### 4.9. HA Affinity Chromatography of the DEAE SPI Pools

Four SPI pools from DEAE anion exchange were applied to a column of immobilised HA and the column eluted with 3 bed volumes starting buffer (50 mM Tris-HCl pH 7.2). Bound material was eluted with 2 M NaCl in starting buffer. Eluant fractions (5 mL) were collected at a flow rate of 10 mL/h. Aliquots (50 µL) of fractions were monitored for trypsin inhibitory activity. Bound fractions containing SPI activity were subsequently pooled as shown in Figure 3.

### 4.10. Sephadex G100 Gel Permeation Chromatography of HA Affinity Purified SPI Samples

A XK16/100 (1.6 × 98 cm) column of Sephadex G100 was equilibrated in 50 mM Tris-HCl 150 mM NaCl pH 7.2 and the void volume and total volume of the column determined using Dextran blue and *D*-glucose respectively. Samples of the HA affinity purified SPI pool (2.0 mL) were applied to the Sephadex G100 column at a flow rate of 8 mL/h. Eluent fractions (2 mL) were monitored for protein (A280 nm) and trypsin inhibitory activity. Two SPI pools were subsequently collected by pooling fractions 4–9 (SPI pool 1), and fractions 18–25 (SPI pool 2). After one month of storage at 4 °C these samples were re-chromatographed.

### 4.11. Chymotrypsin Affinity Chromatography

SPI samples from HA affinity or Sephadex G100 gel permeation chromatography were applied to a column of immobilised chymotrypsin (1 mL bed volume). The column was washed with 1 M NaCl in 0.5 M sodium acetate, buffer pH 4.0 starting buffer, to remove non-bound material. Bound SPIs were eluted from the column with 3 mM HCl, pH 2.0, into aliquots of 1 M Tris free base to adjust the pH of the collected fraction to 7.4. 

### 4.12. Concanavalin A Lectin Affinity Chromatography of SPI Samples

SPI samples from HA affinity or Sephadex G100 gel permeation chromatography were applied to a Concanavalin A Sepharose 4B column (3.5 × 1.5 cm) in 50 mM Tris, 200 mM NaCl buffer pH 7.2 starting buffer containing 1 mM CaCl_2_, 1 mM MgCl_2_, and 1 mM MnCl_2_, at room temperature. The column was washed with 10 bed volumes of starting buffer to remove non-bound material. Bound SPIs were eluted with a linear gradient of 0–0.2 M methyl-α-d-glucopyranoside in starting buffer. The ovine AC SPIs eluted at ~0.07 M methyl α-d-glucopyranoside.

### 4.13. Detection of Active SPIs Using Biotinylated Trypsin and Affinity Blotting

SPI samples were electrophoresed on Novex 4–20% SDS-PAGE gradient gels then transferred to 0.45 µm nitrocellulose membranes in 12 mM Tris, 96 mM glycine, pH8.3, 20% *v*/*v* methanol transfer buffer for 2 h at 200 mA [24]. Nitrocellulose membranes were blocked overnight in 20 mM Tris-HCl 500 mM NaCl pH 7.2 containing 0.1% *v*/*v* Tween 20 (TBS-Tween) then rinsed in TBS-Tween prior to incubation with biotinylated trypsin (bT, 0.5 µg/mL) in TBS-Tween for 1 h at room temperature. The membranes were then washed again in TBS-Tween then incubated for 1h with avidin-alkaline phosphatase 1/1000 dilution. After a final wash the membranes were incubated in NBT/BCIP substrate in 0.1M Tris-HCl pH 9.5 colour development buffer for 20 min.

### 4.14. Development of SPI Western Blots with Antibodies to Chondroitin-4 Sulfate Stub Epitope, Bikunin, TSG-6 and α1-Microglobulin

SPI samples which had been subjected to electrophoresis on 4–20% PAG gradient gels in 0.1M Tris 0.1 M Tricine pH 8.4 and transferred to nitrocellulose membranes in 12 mM Tris, 96 mM glycine, pH 8.3, 20% *v*/*v* methanol transfer buffer for 2 h at 200 mA were blocked overnight in 2% *w*/*v* BSA in 50mM Tris-HCl pH 7.2 containing 0.15 M NaCl (Tris-saline). 

Chondroitinase ABC 0.05 U/mL was added to the 2-B-6 (+) blots and these were incubated at 37 °C to generate the 2-B-6 (+) stub epitopes from the CS linkage regions during the membrane blocking step. All other blots were blocked overnight in 2% BSA in Tris-saline at 4 °C. The blocked blots were incubated with a 1/10 dilution of 2-B-6 hybridoma medium, anti-bikunin (10 µg/mL), anti-α1-microglobulin (5 µg/mL) or anti-TSG-6 antibody (5 µg/mL). After incubation overnight at 4 °C the blots were washed in Tris-saline then anti-rabbit or anti-mouse IgG alkaline phosphatase conjugated secondary antibodies (1/1000 dilution) were added at room temperature for 2 h. After washing again, the blots were incubated with NBT/BCIP in alkaline phosphatase development buffer. Colour development was terminated after 45 min.

### 4.15. Sulfated GAG Analysis

The sulfated GAG content of eluent fractions was used as an index of their proteoglycan contents. Sulfated GAG was determined by reaction with the metachromatic dye 1,9-dimethyl methylene blue as indicated earlier [143].

### 4.16. Cartilage Histological Processing and Confocal Microscopy

Full thickness bovine cartilage was harvested from the tibial plateau of a 2-year-old steer, fixed in 10% neutral buffered formalin for 24 h, dehydrated through sequential alcohol washes and xylene and embedded in paraffin. Cartilage sections (7 µm) were cut using a microtome, attached to positively charged microscope slides and de-waxed in xylene and then rehydrated, through graded ethanol washes then blocked with normal donkey serum (1:20 dilution) for 30 min at room temperature. The sections were digested with proteinase K (Dako) for 20 min rinsed then incubated with anti-lubricin MAb (3A4; 2 µg/mL) anti-versican MAb (12C5, 5 µg/mL) or biotinylated HABP (5 µg/mL) in 50 mM Tris-HCl pH 7.2 0.15 M NaCl overnight at 4 °C. The primary reagents were then detected with Alexa 488 conjugated secondary Ab (1/1000 diln) to mouse IgG or FITC-streptavidin (10 µg/mL) for 8 h, washed in TBS and mounted under coverslips in Vectashield. Propidium iodide was used to stain cell nuclei in the bHABP and lubricin sections. Fluorescently stained tissue sections were visualised using a Leica TCS SP2 AOBS laser scanning confocal microscope (Leica, Heidelberg, Germany) using a 40× oil immersion objective. The slides were scanned using excitation and emission settings for Alexa 488 (Ex max: 488; Em max: 520) Z-stacks of 8-bit ‘optical sections’ (512 × 512 pixels) were taken through the full cartilage thickness at 0.4 µm increments. ‘Maximum Intensity’ reconstructions were prepared from the image stacks using Leica Confocal Software (Leica, Heidelberg, Germany). 

### 4.17. Statistical Analyses

Protease inhibitory activity data were tested for significance using the Kruskal Wallace equality-of-populations rank test in the first instance. If significance was found (*p* < 0.05), samples were compared using the Wilcoxon rank-sum test. Within each parameter, the Benjamini-Hochberg post-hoc test was used to correct for false positives. All statistical analyses were performed using STATA 15 statistical software (Release 14, StataCorp LP, College Station, TX, USA). Each analysis was based on six replicates and the experiment was repeated on at least two further samples. Data was considered significant when test data vs control data groups displayed *p* < 0.05.

## 5. Conclusions

(1) A retrospective analysis of earlier ovine AC and IVD studies combined with data generated in the present study shows that ovine AC and IVD contain SPIs synthesised by their resident cells related to ITI/bikunin which are released from the bikunin precursor protein and display potent inhibitory activity against leucocyte elastase, cathepsin G, trypsin, chymotrypsin, plasmin and kallikrein.

(2) A survey of the literature shows that ITI/bikunin are multifunctional KPI domain proteins with a diverse range of biological properties beyond protease inhibition that are beneficial to tissues in terms of prevention of bacterial, fungal and viral infection, modulation of innate immunity in host-defense and have cell proliferative and anti-tumour properties. The dual function of some KPI domains in protease inhibition and blocking of ion-fluxes in voltage gated ion-channels indicate KPIs may be of therapeutic value in neural disorders. Furthermore, the embedded CS-D motifs in the CS chains of bikunin may equip it with important cell regulatory and neuritogenic properties which need to be explored further.

(3) Studies in other tissues show that HC chain transfer from ITI provides matrix stabilisation in lung tissue, supports oocyte expansion which is essential in fertilisation however in some cases the ITI-HCs may affect HA turnover adversely, deleteriously effecting physiological processes in cystic fibrosis, diabetes, asthma, hyperglycemia and atherosclerosis.

(4) We have demonstrated components in the cartilage surface that ITI is known to be interactive with in vitro. Such interactions in-situ may result in the localisation of ITI in the surface regions of articular cartilage and would be expected to have important protective roles to play which preserve joint functional properties. Furthermore, by analogy with other tissues, transfer of ITI-HCs by TSG-6 to HA or versican may further stabilise this functionally important surface region of articular cartilage.

(5) Development of biotinylated trypsin is a useful sensitive detection system capable of detecting low ng levels of active KPI domains in bikunin and ITI species demonstrating a further functional dimension to ITI proteoglycan species. This detection system can be used along with conventional immunohistological detection systems and demonstrates the ITI species are biologically active.

## Figures and Tables

**Table 1:** Serine proteinase inhibitory (SPI) activities of the Bikunin Kunitz protease inhibitor (KPI) domains and bovine pancreatic trypsin inhibitor (BPTI).

Proteinase	Substrate *	Mean Relative Inhibitory Activity by 1 Unit of SPI ** (% Inhibition) ± SD (*n* = 6)		
		Sheep Kunitz Domain 1	Sheep Kunitz Domain 2	BPTI
Porcine pancreatic trypsin	ZAPNA	95 ± 2.61	97 ± 1.47	96 ± 3.54
Porcine Pancreatic chymotrypsin	AAVANA	52 ± 4.05	55 ± 2.86	53 ± 3.73
Human Leucocyte elastase	SAAVNA	67 ± 4.79	69 ± 3.06	78 ± 4.07
Human Leucocyte cathepsin G	SAAPPNA	18 ± 7.09	25 ± 5.01	28 ± 4.03
Porcine Kallikrein	VLANA	55 ± 8.84	86 ± 4.41	92 ± 5.68
Porcine Plasmin	VLLNA	51 ±4.84	85 ± 1.94	88 ± 8.84

** One unit of inhibitory activity was defined as the amount of SPI required to give 50% inhibition of 0.2 μg of active site titrated trypsin. Each proteinase (0.2 µg) was incubated with one unit of SPI and residual enzyme activity assessed relative to enzyme samples incubated in the absence of inhibitor using the indicated substrates. * Abbreviations: ZAPNA: CBZ-Arg-4 NA; AAVANA: Ala-Ala-Val-Ala 4-NA; SAAVNA: *N*-Succinyl-Ala-Ala-Val-4NA, SAAPPNA: *N*-Succinyl-Ala-Ala-Pro-Phe 4-NA; VLANA: d-Val-Leu-Arg 4NA; VLLNA: d-Val-Leu-Lys 4NA; SPI: Serine proteinase inhibitor; KPI: Kunitz protease inhibitor.

**Table 2:** Serine proteinase inhibitory activities of the Bikunin Kunitz KPI domains and BPTI.

Proteinase	Statistical Significance	
	KPI-1 vs. BPTI	KPI-2 vs. BPTI
Porcine pancreatic trypsin	NSD	NSD
Porcine Pancreatic chymotrypsin	NSD	NSD
Human Leucocyte elastase	NSD	NSD
Human Leucocyte cathepsin G	KPI-1 < BPTI; *p* < 0.05	NSD
Porcine Kallikrein	KPI-1 < BPTI; *p* < 0.05	NSD
Porcine Plasmin	KPI-1 < BPTI; *p* < 0.05	NSD

## Abbreviations

AC	articular cartilage
ITI	inter-α-trypsin inhibitor
Pre-α-TI	pre-α-trypsin inhibitor
SPI	serine proteinase inhibitor
KPI	Kunitz protease inhibitor
CS	Chondroitin sulfate
HC	heavy chain (ITI)
OA	osteoarthritis
RA	rheumatoid arthritis
HA	hyaluronan
SLPI	secretory leucocyte proteinase inhibitor
TOM	Trappin ovine molecule
bT	biotinylated trypsin
TSG-6	Tumour necrosis factor-stimulated gene-6
GAG	glycosaminoglycan
KPI	Kunitz protease inhibitor
KS	Kaposi sarcoma
UTS	urinary trypsin inhibitor
BPTI	basic pancreatic trypsin inhibitor
IVD	intervertebral disc
ChD	chondrodystrophoid
Non-ChD	non-chondrodystrophoid
